# Factors influencing delays in the diagnosis and treatment of bipolar disorder in adolescents and young adults: systematic scoping review

**DOI:** 10.1192/bjo.2026.11049

**Published:** 2026-05-25

**Authors:** Alexander Levit, Frederick Lam, John-Jose Nunez, Emma Morton, Kamyar Keramatian

**Affiliations:** Department of Psychiatry, The University of British Columbia, Vancouver, Canada; School of Psychological Sciences, Monash University, Clayton, Australia

**Keywords:** Bipolar type I or II disorders, mental health services, diagnostic medicine, child and adolescent psychiatry, systematic review

## Abstract

**Background:**

Care for bipolar spectrum disorders is often delayed, and these delays are associated with a poorer prognosis. However, little is known about the specific factors that contribute to these delays. Bipolar disorder onset peaks in adolescents and young adults, where barriers to care may be distinct from younger or older populations.

**Aims:**

To identify the available evidence on factors contributing to delays in care for adolescents and young adults with bipolar disorder.

**Method:**

We performed a pre-registered systematic search of the literature on delays in the care of 13- to 24-year olds diagnosed with bipolar disorder. Our search yielded *n* = 5991 unique articles published between 2000 and 2025. Two independent reviewers screened abstracts and full texts for eligibility according to *a priori* inclusion criteria. Findings from included studies (*n* = 27) were summarised in a narrative synthesis, organised according to patient, disease and systemic factors within the Model of Pathways to Treatment.

**Results:**

Findings were limited to observational levels of evidence. There was a relative paucity of research in the appraisal and help-seeking intervals. Some factors in delays to care were consistently identified across multiple studies. However, there were also contradictions or a lack of replication around identified factors.

**Conclusions:**

Research in this area has been declining in the past decade despite contradictory findings and ongoing significant delays in bipolar disorder care. Factors contributing to delays in bipolar disorder care can be effectively organised according to appraisal, help-seeking, diagnostic and pre-treatment intervals. This enables a systematic approach to identifying areas in need of quality improvement and further research.

Bipolar spectrum disorders include complex and severe psychiatric conditions characterised by recurrent episodes of depression and mania or hypomania. Despite its relatively high prevalence and large disability burden, bipolar disorder often goes unrecognised and untreated for several years. A recent meta-analysis identified a median delay of 7 years from first mood episode to diagnosis of bipolar disorder.^1^ Delays have been associated with various negative outcomes,^2^ including crucial age-specific developmental tasks,^3^ greater severity and frequency of mood episodes,^4^ poorer response to treatment,^5^ higher number of hospital admissions,^6^ higher number of comorbidities^7,8^ and elevated risk of suicide.^7,9,10^ In addition, delay in the diagnosis of bipolar disorder has been shown to be associated with significantly higher healthcare costs,^11^ as well as higher indirect costs owing to work loss.^11,12^

Despite the significant patient and societal burden of bipolar disorder, the factors contributing to delays in care are not well understood. A depressive polarity at the onset of bipolar disorder is often presumed to contribute to much of the delay in care caused by diagnostic challenges.^13^ However, available data indicates that depressive polarity at onset does not fully account for delays in diagnosis.^13,14^ Additionally, there are likely many factors in the periods preceding and following diagnosis that may contribute to delays in accessing guideline-recommended care, including lack of societal awareness, stigma, treatment side-effects and denial or lack of insight.^15^ These factors may not be readily quantifiable, such as in meta-analyses,^1,2^ and may first require qualitative characterisation. Addressing these factors can help guide early intervention research and efforts, such as educational interventions for at-risk youth,^16,17^ ultimately reducing the burden of untreated symptoms on individuals, families and healthcare systems. However, these factors have not been systematically reviewed in the literature to date.

The Model of Pathways to Treatment framework by Scott and colleagues has identified a conceptual framework to organise the intervals that lead up to evidence-based medical treatment.^18,19^ This framework has been utilised in other medical disciplines, most commonly in oncology,^20–22^ but has not been applied to psychiatric conditions. According to this multidimensional framework, the total time from the onset of mood symptoms until treatment initiation is divided into four quasi-sequential intervals:

- appraisal interval: defined as the time from the onset of mood symptoms to perceiving a reason to discuss such symptoms with a healthcare professional;
- help-seeking interval: describes the time from perceiving a reason to discuss mood symptoms with a healthcare professional to the first consultation regarding those symptoms;
- diagnostic interval: represents the time between the first appointment with a healthcare professional and receiving the accurate diagnosis of bipolar disorder;
- pre-treatment interval: describes the time between accurate diagnosis of bipolar disorder and initiation of effective, evidence-based interventions.

This scoping review aimed to identify factors involved in the delayed diagnosis and treatment of bipolar disorder in adolescents and young adults. To facilitate a systematic review of potential factors, we organised findings according to the intervals of the Model of Pathways to Treatment and categorised them as patient, disease or systematic factors. We opted to focus on the adolescent and young adult demographic because this age group captures the peak onset of bipolar disorder (estimated to be 17.5 years),^23^ a period associated with particularly long delays in diagnosis and a severe clinical course. In doing so, we aimed to focus on the unique factors that contribute to the delayed diagnosis and treatment of bipolar disorder during a critical period of development.^19,24^

## Method

Methods for our systematic scoping review are detailed in a previously published protocol,^25^ which was also registered before data extraction on Open Science Framework (identifier QCUG7). In brief, this scoping review was conducted according to the six-stage methodological framework outlined by Arksey and O’Malley,^26^ reported using the Preferred Reporting Items for Systematic Reviews and Meta-Analyses guideline for scoping reviews (see checklist in the Appendix). We also incorporated recommendations by Levac and colleagues^27^ to enhance the Arksey and O’Malley methodological framework.

### Search strategy and selection criteria

The search strategy adhered to the published protocol in full. We queried the Medline (Ovid), EMBASE, PsycINFO and CINAHL databases, using the search terms detailed in Supplementary Table 1, on 9 May 2025. Our *a priori* study inclusion criteria were:

- age: studies of adolescents and young adults with onset of mood symptoms or study enrolment at a mean age of 13–24 years;
- disease: diagnoses of bipolar disorder (including bipolar disorder type 1, bipolar disorder type 2, bipolar disorder not otherwise specified, cyclothymic disorder or unspecified/other specified bipolar and related disorder). Studies that include mixed populations where more than 80% of participants had bipolar disorder, or where bipolar disorder-specific data could be extracted, were also included;
- concept: included data on patient, disease and healthcare system-provider factors related to the components of delay in the diagnosis and treatment of bipolar disorder (see below for further details and Supplementary Table 1 for specific terms);
- publication type: primary qualitative and quantitative research published in peer-reviewed journals;
- publication date: we reviewed literature published from 2000 through the query date of 9 May 2025 to focus on contemporary studies using diagnostic criteria for bipolar disorder.

We excluded grey literature and non-English articles because of resource limitations. On full-text review, we also excluded studies that did not have informative data on delays in care, i.e. no pertinent negative nor positive associations between any investigated factors and the pathway to bipolar disorder treatment. There were no exclusions based on geography nor clinical setting.

The search terms required that articles include terminology pertaining to bipolar disorder, our target age range and terms potentially related to delays in seeking and receiving care (Supplementary Table 1). A conceptual framework based on the Model of Pathways to Treatment by Scott and colleagues informed our conceptual search terms.^18,19^ Within each interval, we considered patient, disease and systemic factors to ensure a systematic review of the literature.^18,19^ Such an approach would also allow us to identify potential gaps in the literature. Patient factors included demographics such as family history and socioeconomic factors. Disease factors included aspects of bipolar disorder presentation, comorbidities and other disease related factors. Systemic factors included clinician, health system and other care provision factors. Although there are several issues with referring to bipolar disorders as diseases rather disorders, we felt it to be appropriate to apply the conceptual category of ‘disease factors’ to bipolar disorder, and favoured maintaining consistency with the terminology utilised in the established framework.^18^ Similarly, we used the terms gender, sex, race and ethnicity as originally reported in cited studies in our data extraction and narrative synthesis.

### Study selection, data extraction and synthesis

**Fig. 1:**
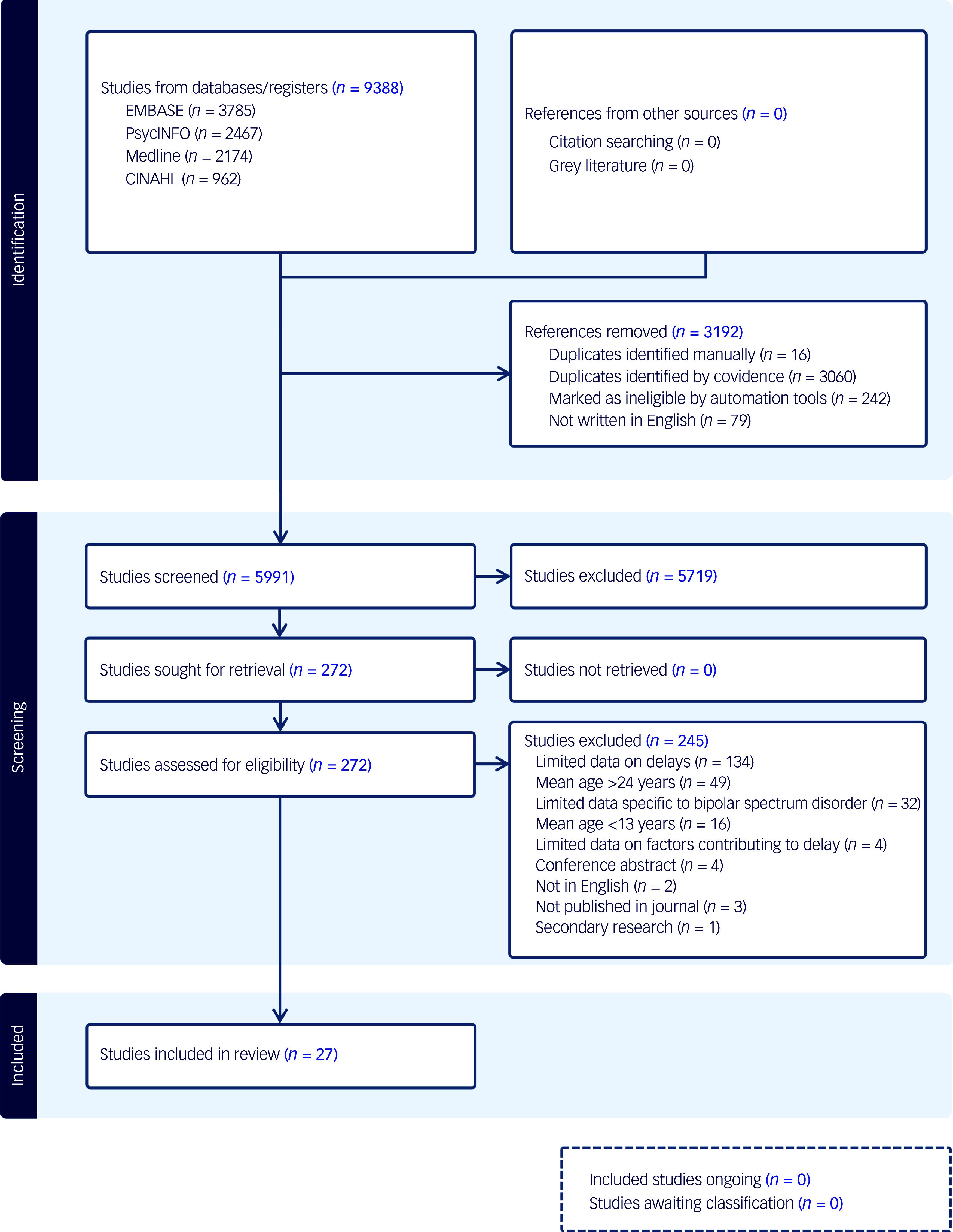
PRISMA flow diagram.

The number of articles screened, assessed for eligibility and selected are summarised in Fig. 1. We used Covidence, a web-based collaborative software platform (Veritas Health Innovation, Melbourne, Australia; https://www.covidence.org), to generate consensus independent reviewer consensus on study selection. Two of three reviewers (A.L., J.-J.N., K.K.) independently screened each title and abstract retrieved by the literature search. Screening disputes were resolved in discussion with a third independent reviewer (A.L., J.-J.N., K.K.). Full texts were retrieved for all studies that were included based on title and abstract screening. Each of these full texts were then also independently assessed by two out of three reviewers (A.L., J.-J.N., K.K.) for eligibility. Disputes around final inclusion were again resolved in discussion with a third independent reviewer (A.L., J.-J.N., K.K.). Extracted data from the included studies was reviewed by two out of three reviewers (A.L., E.M., K.K.). If relevant data were not available in the published manuscript, requests for the missing information were sent to the corresponding authors via email.

As the goal of this scoping review is identify the available relevant literature and gaps in investigation, rather than any specific estimations of an effect or recommendations, a formal Grading of Recommendations Assessment, Development, and Evaluation (GRADE) assessment was not pursued.

### Consulting with stakeholders

Clinicians, researchers, and individuals with lived experience were consulted regarding the relevance, design and preliminary findings at the Canadian Psychiatric Association 2024 Annual Conference (Montreal), the International Society for Bipolar Disorders 2024 Annual Conference (Reykjavik) and locally, with the support of the Michael Smith Health Research BC Convening & Collaborating Program.

## Results

### Search yield

The systematic search yielded 5991 unique publications that were included for initial abstract screening (Fig. 1). Of those, 272 were included for full-text review and full texts were retrieved for all of these studies. Of the 245 studies excluded following full-text review, 55% were primarily excluded because of a lack of quantitative or qualitative data on delays in the appraisal, help-seeking, diagnosis or treatment intervals. Other studies were excluded because the study sample had an age at illness onset or study participation that was older (20%) or younger (7%) than our target range of 13**–**24 years; 13% of studies did not report data on a study population or a subgroup of which at least 80% were diagnosed with a bipolar spectrum disorder. The other 5% of studies were excluded because they were not primary research, not journal articles, not published in English or reported data on delays but featured no exploration of factors that could be associated with these delays.

**Fig. 2:**
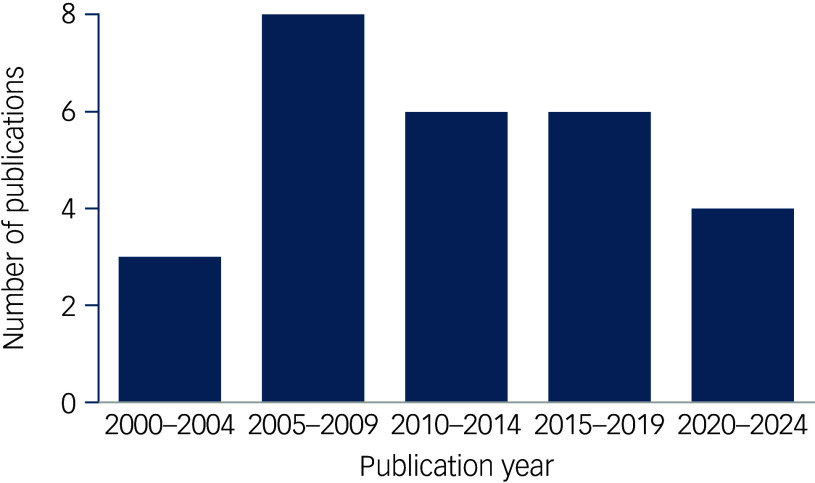
Included studies sorted by publication year.

The final yield of studies that met all inclusion criteria was 27 (detailed in Supplementary Table 2). Of these 27 studies, 12 had a primarily cross-sectional design (including chart review and surveys), 7 were cohort studies, 4 were population database studies, 3 were derived from administrative data and 1 was a qualitative study; some studies combined different research designs (detailed in Supplementary Table 2). In our initial search yield, the number of publications dramatically increased year over year in the time period that was included in our search. However, from the studies that met all inclusion criteria, there was an unexpected peak in the 2005–2009 period, after which there were fewer publications that met our criteria (Fig. 2). Of the included studies, three studies were multinational, and overall, ten studies derived data from USA samples, eight from European samples, three from Oceania samples, two each from Canadian and Indian samples, and one each from Egypt and Oman.

A narrative summary of findings from the included 27 studies follows below, identifying factors that were or were not found to be associated with delays in the pathway to treatment. Factors that were significantly associated with contributing delays in the pathway to treatment are also summarised in Table 1. Of the 27 included studies, 13 publications featured data relevant to the diagnostic interval, 12 to the pre-treatment interval, 5 to the help-seeking interval and only 2 featured data relevant to the appraisal interval; in terms of factor categories investigated, 19 of the 27 studies reported on patient-related factors; 17 reported on disease-related factors and 11 reported on systemic factors (Supplementary Table 3).

**Table 1:** Summary of factors identified in included studies that were associated with delays in the pathways to treatment according to individual and related factors (including patient, family and socioeconomic factors), disease and related factors (including manifestations of bipolar disorder and comorbidities), and systemic factors (including clinician and health system factors)

Appraisal interval	
Patient	Misattribution of early symptoms to childhood adversity, interpersonal conflict/grief or trauma^28^ and supernatural aetiology^29^
Disease	Misattribution of early symptoms to comorbid substance use disorder^28^
Systemic	–
Help-seeking interval	
Patient	Male gender^30^ Earlier age of illness onset^31^; younger age^a^ ^30,32^ Traditional faith-based healing,^29^ which was associated with lower education^33^ and higher level of functioning^33^
Disease	Hallucinations (associated with traditional faith-based healing)^33^
Systemic	–
Diagnostic interval	
Patient	Earlier age of illness onset^a^ ^31,34,35^; younger age^36–38^ No family history of bipolar disorder^39^ Indigenous/racialised minority status^a^ ^34,40^
Disease	Non-psychotic or non-disabling initial episodes^34^; milder illness severity^37,38^ More psychiatric comorbidities before index diagnosis of bipolar disorder^38^
Systemic	Lack of systematic screening for bipolar disorder^39,41,42^ Lack of access to psychiatrists^43^ or mental health professionals^38^ Non-managed care (USA health system context)^38^
Pre-treatment interval	
Patient	Older age at onset^a^ ^34,37,44–46^ Racialised minority status^a^ ^34,46,47^ Low socioeconomic status^48^ Rural residence (psychotherapy, but not pharmacotherapy)^46^ Low perceived helpfulness of treatment^47^
Disease	Bipolar disorder type 2^a^ ^34,49,50^ Early episodes without disability^34^ or psychosis^a^ ^34,45^ Comorbid substance use disorder^a^ ^46–48^ or attention-deficit hyperactivity disorder^a^ ^45,46,48^
Systemic	Not receiving care from a psychiatrist^45,46^ Not having required in-patient care^a^ ^45,46^ Fewer out-patient visits^46^ Fewer non-medication-related mental health encounters^47^ Inconsistent diagnosis^37^ Country of residence (USA > Europe)^44^

For non-significant trends or pertinent factors that were found to be not associated with delays, see main text.

a. Factor was inconsistently associated with delays.

### Appraisal interval

Only two qualitative studies contained findings specific to the appraisal interval. The first reported that some delay of appraisal could result from parental misattribution of early symptoms of bipolar disorder to reactions to childhood adversity, family conflict, grief and trauma.^28^ Similarly, early symptoms of bipolar disorder were also misattributed to comorbid substance use disorder (SUD).^28^ Parental self-blame for their child’s symptoms also emerged as a common experience in this study. A qualitative study from India found that in comparison to major depressive disorder (MDD), bipolar disorder was more likely to be attributed to supernatural punishment by caregivers.^29^

### Help-seeking interval

Five studies were pertinent to the help-seeking interval, of which five included data on patient factors, two on disease factors and one on systemic factors.

Patient factors identified as contributing to delays in the help-seeking interval included male gender and younger age at onset. However, one study demonstrated a complex association with occupational status and one study found no association with indigenous status. A study of Omani students diagnosed with bipolar disorder identified male gender as having a reduced likelihood of contact with care services.^30^ This study also reported that 14- to 16-year olds were less likely to have had contact with care services than those over the age of 17 years. In a cross-sectional study of Finnish youth, having a first mood episode of any polarity before age 18 years was associated with a greater time to first contact with psychiatric care.^31^ This was true for both time from first symptoms (11.9 *v*. 7.2 years) and first episode (10.2 *v*. 5.5 years). Similarly, in a Canadian population study, 15- to 18-year olds diagnosed with bipolar disorder were less likely to report a lifetime contact with mental health services than 19- to 24-year olds (46 *v*. 60%); a similar pattern was observed in those who reported a comorbid SUD (23% *v*. 46%; SUD itself was not analysed as a factor in service use).^32^ However, this study demonstrated an interaction between age and occupational status: the global age-related trend was similar among full-time employees (lifetime contact with mental health services reported in 48% of 15- to 18-year olds and 82% of 19- to 24-years olds who were full-time employees), a major inversion was observed in full-time students (81% of 15- to 18-year olds and 23% of 19- to 24-year olds who were full-time students).^32^ In New Zealand, indigenous status was not associated with differences in contacting mental health services for affective psychosis.^40^

The qualitative study of caregivers beliefs about bipolar disorder as compared with MDD in India found that because of the frequent attribution of bipolar disorder to supernatural punishment, more time and other resources would be spent on pursuing traditional faith healing practices.^29^ Similarly, a study from Egypt found that 41% of study participants sought advice from traditional healers (defined as religious or spiritual counsellors), of whom 62% did so before contacting psychiatric services. This was assumed to delay appropriate care; however, an important limitation of this study was that this assumed delay was not explicitly measured.^33^ In this study, seeking help from traditional healers was associated with lower educational level but higher global assessment of functioning; the authors’ interpretation was that greater impairment owing to illness would push individuals to seek psychiatric support.^33^ Gender, age and family history were not associated with seeking help from traditional healers.^33^ This was the only included study that investigated help-seeking in relation to disease factors within the appraisal interval. It found that a history of hallucinations was associated with seeking help from traditional healers (as defined above), whereas having psychiatric comorbidities was associated with a negative history for seeking help from traditional healers. There was no association with accessing traditional healer with bipolar disorder type, episode polarity, episode severity, number of episodes or the presence of delusions.^33^ No other studies were found that explored how help-seeking was associated with neither disease factors nor systemic factors.

### Diagnostic interval

Thirteen included studies were pertinent to the diagnostic interval, of which ten had data on patient factors, eight had data on disease factors and six had data on systemic factors.

Patient factors identified as contributing to delays in the diagnostic interval included age (although this finding was inconsistent), an absence of family history, and indigenous or ethnic minority status.

Earlier age at illness onset was associated with delayed diagnosis in two studies.^34,35^ However, in a study that offered a more detailed breakdown of the trajectory to diagnosis, earlier age at onset was a factor in the help-seeking interval, but it was not associated with the time from first contact to diagnosis, i.e. the diagnostic interval specifically.^31^ This degree of chronological resolution was not provided in the other two studies, wherein the role of age at onset in the help-seeking interval may confound interpretation of their results. In contrast to age at onset, younger age at time of initial contact was associated with a lower likelihood of diagnosis on initial contact in a Danish population study.^36^ In the USA context, age was not associated with consistency of insurance claims related to a new diagnosis of bipolar disorder (i.e. diagnostic stability).^37,38^

Gender was not associated with delays in diagnosis^34,51^ or consistency of diagnosis.^37,38^ Race was also not associated with delays in diagnosis in a USA study.^34^ However, in New Zealand, indigenous status predicted lower rates of bipolar disorder type 1 diagnosis and higher rates of a diagnosis of schizophrenia following initial presentation of affective psychosis.^40^ Although the data could not confirm that this was attributable to missed recognition of bipolar disorder, the authors considered it the most likely explanation given that other studies have shown similar rates of mood disorders in Maori and Non-Maori patients, as well as underrecognition of mood symptoms and bipolar disorder in other racial or ethnic minorities.^40^ One small cross-sectional study found that absence of a family history of bipolar disorder was the primary risk factor for underdiagnosis of bipolar disorder.^39^

Key disease factors identified as contributing to delays in the diagnostic interval included absence of psychosis and subjective functional impairment. In one cross-sectional study, the average time to diagnosis more than doubled for individuals whose initial mood episodes were non-psychotic and were subjectively not impairing in terms of academic/occupational or social functioning.^34^ Whether illness onset was acute or insidious (recalled subjectively by study participants) had no association with delaying diagnosis.^34^ However, greater illness severity was associated with improved diagnostic stability as inferred from continuous insurance claims.^37,38^ Similarly, a greater number of psychiatric comorbidities made at the time of the initial bipolar disorder diagnosis was associated with a more continuous pattern of insurance claims for bipolar disorder.^38^ In contrast, a greater number of psychiatric diagnoses before an index diagnosis of bipolar disorder was associated with a lower degree of claims continuity.

The included studies did not provide clear data demonstrating an association between polarity at illness onset and time to diagnosis. However, this association was inferred by the authors of one study that showed a median onset of depressive symptoms and episode occurring at 18 and 21 years, in contrast to a median onset of manic symptoms and episode at 21 and 24 years of age, respectively.^35^ This study investigated the diagnostic history of participants diagnosed with either bipolar disorder or schizoaffective disorder. Over half were diagnosed with another condition, most commonly depressive disorders, for a mean duration of 7.6 years before the diagnosis was revised. Similar inferences about a depressive polarity at onset delaying recognition and diagnosis of bipolar disorder were made in two Spanish studies.^43,52^ These studies both showed a high prevalence of depressive symptoms preceding diagnosis. They also observed either a significant drop of depressed mood and anhedonia^43^ or an increase of grandiosity^52^ at the time when the diagnosis was made.

The included studies did not find a clear association between bipolar disorder type and delays in the diagnostic interval. Only one study identified that, compared to patients with bipolar disorder type 1, there was a significantly poorer diagnostic agreement between referring general practitioners and consulting psychiatrists for patients with bipolar disorder type 2.^53^ This difference between bipolar disorder types was not significant in the 18- to 25-year-old age group, although there was a non significant trend for better diagnostic agreement in older age groups.^53^

Regarding clinician and systems factors in the diagnostic interval, lack of systematic evaluation and lack of access to psychiatry was identified as contributing factors. One cross-sectional study from 2005 concluded that lack of systematic interviewing or screening for bipolar disorder was a leading factor for missed diagnoses of bipolar disorder in over half of referred youth initially diagnosed with MDD, although this attribution was not specifically measured.^41^ Similar conclusions were made in another cross-sectional study from 2007 that a systematic diagnostic interview diagnosed bipolar disorder type 2 in just under one out of five youth originally diagnosed with MDD.^42^ In a 2011 cross-sectional study of youth diagnosed with bipolar disorder using a systematic diagnostic interview, one out of three were previously diagnosed with MDD and just over one out of three were diagnosed with attention-deficit hyperactivity disorder (ADHD), whereas only one youth with an initial diagnosis of bipolar disorder was revised to MDD.^39^ Of relevance to these findings, suspicion for bipolar disorder as the reason for referral and diagnosis of bipolar disorder by a psychiatrist was found to be poor in one clinic in Montreal, with an interrater *κ*-value of 0.3, although data on delayed diagnosis was not available for association with referral reason-diagnosis agreement.^53^ In a Spanish cross-sectional study, delays in getting to see a psychiatrist was identified as the primary factor for delays in diagnosis. Concordantly, in the USA context, consistent insurance claims related to a new diagnosis of bipolar disorder (i.e. diagnostic stability) were more likely for youth who received an index diagnosis from a mental health professional than other professionals, and among those who received managed care instead of fee-for-service models.^38^

### Pre-treatment interval

Ten included studies were pertinent to the pre-treatment interval, of which seven had data on patient factors, eight had data on disease factors and five had data on systemic factors. In general, we found it challenging to reliably map the pre-treatment interval onto cross-sectional study designs, as ‘treatment delay’ often incorporated elements of the appraisal, help-seeking and diagnostic intervals, unless these stages were parsed out. Where relevant, we note when results cannot be reliably attributed to the pre-treatment interval.

There were some contradictory findings on the role of age in the pre-treatment interval. In one cross-sectional study, earlier age at onset was associated with a greater time to introduction of a mood stabiliser, although the study design would not have allowed for investigation into the time period specific to the pre-treatment interval.^34^ Data from the Stanley Foundation Bipolar Treatment Outcome Network (SFBN) found that individuals with symptom onset in adolescence (aged 13**–**18 years) had a greater delay to first treatment than those who had onset later in life.^44^ However, this data was also likely not specific to the pre-treatment interval. In contrast, study designs that enabled investigation of time periods more specific to the pre-treatment interval found the opposite. Insurance claims data that evaluated treatment after a new diagnosis revealed that older youth who saw a psychiatrist leading up to index diagnosis of bipolar disorder were less likely to receive guideline concordant care than younger youth.^45^ Similarly, another study of insurance claims data showed that older youth were less likely to ever fill a prescription and to have a continued pattern of prescription filling.^37^ This was not replicated in a third study of insurance claims, which found that age was not associated with guideline concordant pharmacotherapy or metabolic screening.^46^ However, this study did find that older age was associated with lower rates of guideline-concordant psychotherapy and monitoring for side-effects and drug levels.^46^

Socioeconomic factors were also identified as playing an important effect on the pre-treatment interval, although findings regarding the role of patient ethnicity or race were mixed. Low socioeconomic status was associated with non-adherence.^48^ In a USA cohort study that focused on the impact of race on treatment initiation, initial treatment adherence was lower in Black patients than in White patients (56 *v*. 72%).^47^ Black patients were more likely to be prescribed antipsychotic medications (70 *v*. 24%) and for a longer proportion of follow-up than White patients, even without psychosis present.^47^ Rates and duration of antipsychotic prescribing were not accounted for by non-adherence rates; only race and psychosis predicted the rate of antipsychotic prescription. Although subjective evaluations of the helpfulness of treatment was generally associated with better treatment adherence, race was not associated with subjective perceptions of medication helpfulness.^47^ Race was also not associated with antipsychotic dosing or duration of treatment with antipsychotics after remission. Additionally, race was not linked to rates of mood stabiliser prescription in this same study,^47^ a finding also observed in another cross-sectional study.^34^ However, elsewhere, administrative data found that Black, Asian and minority ethnic youth had lower odds (odds ratio 0.7) of receiving guideline-concordant medications than White youth.^46^ Race and ethnicity was not associated with blood-level monitoring, side-effect monitoring, metabolic screening or adjunct psychotherapy. Youth residing in non-urban areas were more likely to receive indicated metabolic screening, but less likely to receive adequate psychotherapy.^46^ Density of residence was not associated with pharmacotherapy, blood level monitoring or side-effect monitoring.^46^ Gender was not associated with receipt of guideline-concordant care.^34,46^

Disease factors were inconsistently linked to treatment delay. One cross-sectional study concluded that neither polarity of first mood episode nor bipolar disorder type was associated with differences in time to treatment.^34^ The absence of an association between bipolar disorder type and time to treatment was also observed in another cross-sectional study.^49^ In contrast, a more recent cohort study showed that the first mood episode for bipolar disorder type 2 was more likely to go untreated within the first 2 years of symptom onset. This may be partly attributable to a later onset of hypomania in bipolar disorder type 2 by 3 years relative to the onset of hypomania or mania in bipolar disorder type 1, or could be related to illness severity, as the first mood episode was treated in an in-patient setting in 45% of patients with bipolar disorder type 1 versus 19% of patients with bipolar disorder type 2.^50^ However, by design, these three studies have poor specificity to the pre-treatment interval. Although cross-sectional data suggested that disability and psychotic features with first mood episodes shortened delay to treatment,^34^ insurance claims data did not support the association with psychotic features at diagnosis and receiving guideline-concordant care.^45^

Comorbidities were often identified as a strong factor in the pre-treatment interval. SUD^47,48^ and ADHD^45,48^ were associated with lower adherence rates. One study contradicted this, noting that comorbid conditions were not associated with receiving appropriate pharmacotherapy, blood level monitoring, side-effect monitoring or metabolic screening, although still identified comorbid SUD (but not ADHD) as lowering the odds of receiving adequate duration of psychotherapy.^46^ Patients who developed heavy cannabis use after onset of manic symptoms had a greater duration of untreated mania, whereas pre-existing heavy cannabis use was not associated with duration of untreated mania. Similar associations were identified with duration of untreated bipolar disorder, but these did not reach statistical significance.^54^

Regarding systemic factors, receiving care from a psychiatrist increased the likelihood of guideline-recommended pharmacotherapy initiation.^45,46^ However, receiving care from a psychiatrist was not associated with drug serum level monitoring, side-effect monitoring, metabolic screening or adjunct psychotherapy.^46^ Primary care visits related to bipolar disorder were associated with higher likelihood of drug serum level monitoring and metabolic screening. However, primary care visits were not associated with appropriate pharmacotherapy, side-effect monitoring or adjunct psychotherapy.^46^

Initial encounter setting, encounter frequency and encounter focus were identified as important factors. In-patient care was inconsistently associated with receiving guideline-recommended pharmacotherapy, with one study finding a doubling of odds for youth who had an in-patient visit,^46^ whereas another study found no association with pharmacotherapy and actually reduced odds of receiving adequate psychotherapy.^45^ Having more out-patient visits increased the odds of receiving appropriate pharmacotherapy and psychotherapy.^46^ A greater number of non-medication-related mental health contacts was associated with better initial treatment adherence.^47^ Similarly, psychotherapy was associated with more continuous use of a mood stabiliser, although it was also associated with delayed initiation of a mood stabiliser.^37^ However, an initial delay in starting a mood stabiliser after diagnosis did not independently predict later continuity of mood stabiliser use.^37^ Otherwise, psychotherapy was not associated with receiving guideline-recommended pharmacotherapy.^45^ A continuous pattern of diagnosis in insurance claims was also associated with greater likelihood of initiating and continuing mood stabiliser treatment.^37^

Youth receiving USA Medicaid through disability rather than poverty were less likely to receive adequate psychotherapy. However, Medicaid eligibility was not associated with pharmacotherapy, drug level monitoring, side-effect monitoring or metabolic screening.^46^ The SFBN network study indicated that residing in the USA was associated with more than a doubling of delay to treatment than in Europe, regardless of polarity at onset.^44^ However, the design of this study may not be specific to the pre-treatment interval and may be confounded by an earlier age at onset in the USA, although no statistical interaction between age and country was found.^44^

## Discussion

This systematic scoping review identified literature on the factors influencing delays in the diagnosis and treatment of bipolar disorder in adolescents and young adults, using the Model of Pathways to Treatment framework. This framework examines how stages from symptom appraisal to treatment initiation are influenced by patient, clinician and systemic factors, and was selected to provide a structured and theory-informed approach to capturing the multiple components of delay along the pathway from symptom onset to treatment.

### Identified factors

Several factors associated with delayed care were replicated across studies. In the diagnostic interval, younger age,^31,34–38^ lack of systematic screening for bipolar disorder by clinicians^39,41,42^ and lack of access to mental health professionals^38,43^ were identified as potentially delaying factors. In the pre-treatment interval, older age,^34,37,44–46^ minority demographics,^34,46,47^ milder illness severity,^34,45^ comorbid SUD^46–48^ or ADHD,^45,46,48^ and not receiving care by a psychiatrist^45,46^ contributed to delays. However, findings were inconsistent. Although findings on age were inconsistent, younger age tended to delay help-seeking and diagnosis, whereas treatment initiation was sometimes delayed in older youth.

### Key gaps in the included literature

Applying the Model of Pathways to Treatment framework helped identify areas requiring further investigation. Significant gaps were identified, particularly in the appraisal and help-seeking intervals. Systemic factors were studied less frequently than patient or disease factors.

The paucity of publications relevant to the appraisal and help-seeking intervals likely reflect the inherent challenge of studying the early stages of disease recognition. Further research focused on these intervals is needed, especially as there may be key modifiable risk factors in these intervals such as awareness and stigma. Qualitative studies have shown that individuals with bipolar disorder hold a broad range of prior awareness, beliefs and attitudes, including self-stigma, about their symptoms and diagnosis, and that these may be associated with clinical outcomes in complex and dynamic ways.^55–59^

We were surprised that none of the included studies clearly identified polarity at illness onset or bipolar disorder type as a contributing factor to delays to care. This contrasts prior findings that depressive onset is associated with longer untreated illness (median 11 *v*. 3.5 years for depressive versus hypomanic or manic first episode) as is bipolar disorder type 2 (median 9**–**11 *v*. 5**–**8 years for bipolar disorder type 2 versus type 1).^14,60^ It has been speculated that patients and their supports may not recognise or report signs of hypomania to clinicians, which could contribute to delays.^13,61–63^ Screening for hypomania and mania in patients presenting with depression may reduce diagnostic delays,^64–67^ but may have limited impact on the appraisal and help-seeking intervals. Some delay is likely inherent to the challenge of identifying depressive episodes as part of a bipolar syndrome before there is any onset or recognition of any hypomanic or manic symptoms.^1,13^ This emphasises the need for improvements in clinical differentiation between unipolar and bipolar depressive episodes. In any case, delays in bipolar disorder diagnosis cannot all be attributed to a depressive polarity at onset, as substantial delays in care have been observed even when initial polarity is hypomanic or manic.^14^

We hypothesised that clinician cognitive biases may also contribute to diagnostic delays, although this was not explicitly investigated in our included studies. An individual’s prior diagnosis of MDD could contribute to an anchoring bias that delays consideration of bipolar disorder. Even after a diagnosis of bipolar disorder is made, the relative prevalence of MDD or a cross-sectional depressive presentation could continue to favour a diagnosis of MDD in the form of an availability bias; a 2008 cohort study of 3119 patients with bipolar disorder did find that over a quarter were misdiagnosed with MDD after already having received a diagnosis of bipolar disorder.^68^ Availability bias may also favour attribution of symptoms to precipitating factors such as comorbid substance use or concurrent interpersonal stressors.^69^ Ongoing controversy surrounding the phenomenology and the validity of the diagnosis of bipolar disorder in youth may also contribute to clinician biases and uncertainty, while also shaping perceptions of the condition in ways that may have implications for stigma and help-seeking.^70,71^

### Limitations

Our methodology did have several limitations. Although identified factors were assigned one interval and one category for the purpose of organising a systematic approach, a more nuanced review of these factors would reveal that they could often be re-conceptualised under different intervals and categories. For example, we favoured a clinical lens and categorised a patient’s socioeconomic context as a patient factor, whereas a more sociological or public health lens might identify this as a systemic factor. Some factors may not neatly fit into one interval only, either inherently or because of study design considerations. Although this framework enables a focus on factors pertinent to any one interval in the pathway to treatment, it is still important to remain aware of how factors influence the entire pathway. Otherwise, a siloed focus could lead to an erroneous dismissal of important factors; for example, although indigenous status was not associated with delays in help-seeking, it may have delayed accurate diagnosis.^40^

A fundamental limitation of our findings is that the modal design of included studies was cross-sectional (12 out of the 27 included studies), whereas longitudinal studies that can better capture the trajectory of symptom recognition, diagnosis and treatment were less common. This carries a significant risk for recall biases, and studies indicate that both manic and depressive episodes are prone to being forgotten by patients.^63,72,73^ In our included studies, cross-sectional studies that did not clearly delineate time from diagnosis to treatment made it difficult to assign factors specifically to the pre-treatment intervals. Administrative data, such as insurance claims, were often very informative in the pre-treatment interval, as they could track time from first diagnosis to sustained initiation of indicated treatment.

Finally, we restricted our search to studies of bipolar disorder with onset between ages 13 and 24 years, to focus on the manifestations and challenges specific to this peak period of bipolar disorder onset. Although intended to maintain a focused age range, this criterion may have led to the exclusion of relevant studies that would inform our understanding of bipolar disorder across a broader developmental spectrum. Findings from this study may not generalise to paediatric, adult or geriatric onset bipolar disorder, which are likely to have distinct challenges across the pathway to treatment that should be explored in future research. If studies identified in our search did not identify age at onset, even if patient indeed had mood episodes begin in this age range, they would not have been included in our analysis. Similarly, studies of systemic factors that affected this demographic but were not specific to this demographic would also not have been included in our review. Because of resource limitations, we also were not able to review grey literature or literature published in languages other than English.

### Other challenges and future directions

Although retrospective studies may be limited by recall bias, prospective studies of high-risk youth could introduce sampling and observer bias, whereas population-based prospective studies would be resource-intensive and still prone to observation bias. These potential biases should be recognised and mitigated where possible, but should not preclude research in these poorly understood components of the pathway to treatment.

Early comorbidities likely complicate all stages of the treatment pathway. Thus, a narrow focus on bipolar disorder-specific psychopathology to identify bipolar disorder may not be sufficient.^74^ Rather, the presence of these non-specific symptoms may alert clinicians to the possibility of either a comorbid bipolar disorder or an underlying trajectory toward bipolar disorder, but this will need to be balanced against the risk of overdiagnosis. Indeed, associated non-specific symptoms have been incorporated into risk calculators that identify youth at risk for developing bipolar disorder.^75,76^ The use of these risk calculators, as well as other clinical tools that identify youth at increased risk,^77^ could be used to identify cohorts that would be well suited for future longitudinal research into the process of symptom appraisal, help-seeking, diagnosis and treatment initiation for bipolar disorder. This approach could reduce recall bias and improve the efficiency of prospective studies.

Our scoping review found a potentially dynamic impact of age, with help-seeking and diagnostic intervals tending to be prolonged in younger age, whereas initiation of recommended treatments tending to be prolonged in older youth; however, this pattern was not explicitly investigated in the studies included in our scoping review. We hypothesise that this may reflect older youth’s relatively more common needs around asserting independence. There is emerging evidence documenting that the balance between support and autonomy is particularly desirable for youth.^78–80^ Future research may wish to investigate the potential role of older youth’s needs around asserting independence around treatment.

Our search yield also showed that after 2009, there was a trend over time for fewer publications meeting our inclusion criteria, even though the number of potentially relevant abstracts identified did grow exponentially over time. Over half of full-text articles screened were excluded on the basis of having limited data on delays. This is concerning given persistent delays in bipolar disorder care. We therefore call for renewed research on delays in bipolar disorder care. Moreover, we recommend that future studies attempt to standardise reporting on time to help-seeking and diagnosis, treatment for depressive symptoms/episodes and manic symptoms/episodes, and meeting bipolar disorder diagnosis criteria. This would help clarify how much adolescents and young adults experience delays because of diagnostic uncertainty versus missed diagnoses.

In conclusion, by applying the Model of Pathways to Treatment framework, our scoping review identified a relative paucity in the appraisal and help-seeking intervals for bipolar disorder in adolescents and young adults. Although there are studies identifying potential barriers in the appraisal and help-seeking intervals, these require replication and further exploration. In the diagnostic interval, younger age, lack of systematic screening for bipolar disorder by clinicians and lack of access to mental health professionals were identified as barriers to care. In the pre-treatment interval, older age, minority demographics, milder illness severity, comorbid SUD or ADHD, and not receiving care by a psychiatrist were identified as barriers to care.

Despite the ongoing burden of delays in the care for bipolar disorder, the available literature on factors contributing to delays remains inadequate. This scoping review highlights some of the reported factors that can contribute to delays at various intervals in the pathway to care for bipolar disorder, and some of these delay factors can cut across intervals. As bipolar disorder are heterogenous, often evolving on a backdrop of the major life transitions of adolescence and early adulthood, longitudinal and prospective approaches are likely to be critical in the research.

## Supporting information

10.1192/bjo.2026.11049.sm001 Levit et al. supplementary material Levit et al. supplementary material

## Data Availability

No new data were created or analysed in this study. However, data extractions and meta-data from included studies that support the findings of this study are available from the corresponding author, K.K., upon reasonable request.

## Supplementary material

The supplementary material is available online at https://doi.org/10.1192/bjo.2026.11049

## Appendix. PRISMA-ScR checklist

**Table:** Preferred Reporting Items for Systematic Reviews and Meta-Analyses Extension for Scoping Reviews (PRISMA-ScR) Checklist

Section	Item	PRISMA-ScR Checklist item	Reported in manuscript section
Title			
Title	1	Identify the report as a scoping review.	Title
Abstract			
Structured summary	2	Provide a structured summary that includes (as applicable): background, objectives, eligibility criteria, sources of evidence, charting methods, results, and conclusions that relate to the review questions and objectives.	Abstract; sources of evidence detailed in methods
Introduction			
Rationale	3	Describe the rationale for the review in the context of what is already known. Explain why the review questions/objectives lend themselves to a scoping review approach.	Introduction
Objectives	4	Provide an explicit statement of the questions and objectives being addressed with reference to their key elements (e.g. population or participants, concepts, and context) or other relevant key elements used to conceptualise the review questions and/or objectives.	Introduction
Method			
Protocol and registration	5	Indicate whether a review protocol exists; state if and where it can be accessed (e.g. a Web address); and if available, provide registration information, including the registration number.	Method
Eligibility criteria	6	Specify characteristics of the sources of evidence used as eligibility criteria (e.g. years considered, language, and publication status), and provide a rationale.	Method
Information sources^a^	7	Describe all information sources in the search (e.g. databases with dates of coverage and contact with authors to identify additional sources), as well as the date the most recent search was executed.	Method
Search	8	Present the full electronic search strategy for at least one database, including any limits used, such that it could be repeated.	Method and Supplementary Table 1
Selection of sources of evidence^b^	9	State the process for selecting sources of evidence (i.e. screening and eligibility) included in the scoping review.	Method
Data charting process^c^	10	Describe the methods of charting data from the included sources of evidence (e.g. calibrated forms or forms that have been tested by the team before their use, and whether data charting was done independently or in duplicate) and any processes for obtaining and confirming data from investigators.	Method
Data items	11	List and define all variables for which data were sought and any assumptions and simplifications made.	Method
Critical appraisal of individual sources of evidence^d^	12	If done, provide a rationale for conducting a critical appraisal of included sources of evidence; describe the methods used and how this information was used in any data synthesis (if appropriate).	Not applicable
Synthesis of results	13	Describe the methods of handling and summarising the data that were charted.	Method
Results			
Selection of sources of evidence	14	Give numbers of sources of evidence screened, assessed for eligibility, and included in the review, with reasons for exclusions at each stage, ideally using a flow diagram.	Results and Fig. 1
Characteristics of sources of evidence	15	For each source of evidence, present characteristics for which data were charted and provide the citations.	Not applicable
Critical appraisal within sources of evidence	16	If done, present data on critical appraisal of included sources of evidence (see item 12).	Not applicable
Results of individual sources of evidence	17	For each included source of evidence, present the relevant data that were charted that relate to the review questions and objectives.	Results
Synthesis of results	18	Summarise and/or present the charting results as they relate to the review questions and objectives.	Results and Table 1
Discussion			
Summary of evidence	19	Summarise the main results (including an overview of concepts, themes and types of evidence available), link to the review questions and objectives, and consider the relevance to key groups.	Discussion
Limitations	20	Discuss the limitations of the scoping review process.	Discussion
Conclusions	21	Provide a general interpretation of the results with respect to the review questions and objectives, as well as potential implications and/or next steps.	Discussion
Funding			
Funding	22	Describe sources of funding for the included sources of evidence, as well as sources of funding for the scoping review. Describe the role of the funders of the scoping review.	Funding

PRISMA-ScR, Preferred Reporting Items for Systematic reviews and Meta-Analyses extension for Scoping Reviews.

a Where sources of evidence (see footnote b) are compiled from, such as bibliographic databases, social media platforms and Web sites.

b A more inclusive/heterogeneous term used to account for the different types of evidence or data sources (e.g. quantitative and/or qualitative research, expert opinion and policy documents) that may be eligible in a scoping review as opposed to only studies. This is not to be confused with information sources (see footnote a).

c The frameworks by Arksey and O’Malley^26^ and Levac and colleagues^27^ and the Joanna Briggs Institute guidance^81,82^ refer to the process of data extraction in a scoping review as data charting.

d The process of systematically examining research evidence to assess its validity, results and relevance before using it to inform a decision. This term is used for items 12 and 19 instead of ‘risk of bias’ (which is more applicable to systematic reviews of interventions) to include and acknowledge the various sources of evidence that may be used in a scoping review (e.g. quantitative and/or qualitative research, expert opinion and policy document).^83^
