# Supplementary material for: Factors influencing delays in the diagnosis and treatment of bipolar disorder in adolescents and young adults: systematic scoping review
Source: BJPsych Open. 2026 May 25;12(3):e143. doi: 10.1192/bjo.2026.11049 (PMC13202609; doi:10.1192/bjo.2026.11049)
Supplement: Levit et al. supplementary material [file S2056472426110497sup001.docx]

**Supplementary Table 1: Systematic search terms**

| **OVID-MEDLINE (R)** (and Epub Ahead of Print, In-Process, In-Data-Review & Other Non-Indexed Citations, Daily and Versions) | |
| --- | --- |
| **Illness**  Terms: 6 | bipolar disorder/ OR "bipolar disorder" OR mania OR manic OR hypomania OR hypomanic |
| AND | |
| **Age group**  Terms: 10 | Adolescent/ OR adolescen* OR "Young Adult/" OR "young adult*" OR Pediatrics/ OR pediatric* OR paediatric* OR youth* OR teen* OR juvenile* |
| AND | |
| **Components of delay**  Terms: 60 | "duration of untreated" OR delayed diagnosis/ OR "delayed diagnosis" OR "delay in treatment" OR "treatment latency" OR "latency to treatment" OR "symptom overlap" OR "symptom recognition" OR "symptom appraisal" OR "symptom attribution" OR "symptom misattribution" OR "symptom perception" OR "symptom awareness" OR belief* OR attitude* OR heuristic* OR "cognitive bias*" OR "coping style*" OR "self regulation" OR "screening" OR "help seeking" OR "information seeking" OR Help seeking behavior/ OR Health literacy/ OR Self-efficacy/ OR "Self-efficacy" OR "self medication" OR "self treatment" OR stigma* OR Health services accessibility/ OR "access to care" OR "access to treatment" OR "access to medication*" OR affordab* OR "waiting time" OR polarity OR underreporting OR under reporting OR "therapeutic alliance" OR clinical competence/ OR "clinical competenc*" OR diagnostic error/ OR "diagnostic error*" OR "diagnostic valid*" OR "diagnostic reliability" OR "diagnostic criter*" OR "diagnostic accuracy" OR "stability of diagnosis" OR "diagnostic stability" OR misdiagnosis OR "Referral and Consultation"/ OR referral* OR consult* OR insight OR Attitude to Health/ OR Patient Compliance/ OR adherence OR compliance OR implementation OR policy |
|  | |
| **OVID-Embase** | |
| **Illness**  Terms: 6 | bipolar disorder/ OR "bipolar disorder" OR mania OR manic OR hypomania OR hypomanic |
| AND | |
| **Age group**  Terms: 10 | Adolescent/ OR adolescen* OR "Young Adult/" OR "young adult*" ORPediatrics/ OR pediatric* OR paediatric* OR youth* OR teen* OR juvenile* |
| AND | |
| **Components of delay**  Terms: 60 | "duration of untreated" OR "delayed diagnosis" OR delayed diagnosis/ OR "delay in treatment" OR "treatment latency" OR "latency to treatment" OR "symptom overlap" OR "symptom recognition" OR "symptom appraisal" OR "symptom attribution" OR "symptom misattribution" OR "symptom perception" OR "symptom awareness" OR belief* OR attitude* OR heuristic* OR "cognitive bias*" OR "coping style*" OR "self regulation" OR screening OR "help seeking" OR "information seeking" OR Help seeking behavior/ OR Health literacy/ OR "Self-efficacy" OR "self medication" OR "self treatment" OR stigma* OR Health Care Access/ OR "access to care" OR "access to treatment" OR "access to medication*" OR affordab* OR "waiting time" OR polarity OR underreporting OR "under reporting" OR "therapeutic alliance" OR clinical competence/ OR "clinical competenc*" OR diagnostic error/ OR "diagnostic error*" OR "diagnostic valid*" OR "diagnostic reliability" OR "diagnostic criter*" OR "diagnostic accuracy" OR "stability of diagnosis" OR "diagnostic stability" OR misdiagnosis OR Patient Referral/ OR Referral* OR Consult* OR insight OR Attitude to Health/ OR Patient Compliance/ OR adherence OR compliance OR implementation OR policy |

|  | |
| --- | --- |
| **EBSCOHost-PsycINFO** | |
| **Illness**  Terms: 6 | DE "Bipolar Disorder" OR "bipolar disorder" OR mania OR manic OR hypomania OR hypomanic |
| AND | |
| **Age group**  Terms: 10 | DE "Adolescent Health" OR adolescen* OR DE "Emerging Adulthood" OR young adult* OR DE "Pediatrics" OR pediatric* OR paediatric* or youth* or teen* or juvenile* |
| AND | |
| **Components of delay**  Terms: 60 | "duration of untreated" OR "delayed diagnosis" OR "diagnostic delay" OR "delay in treatment" OR "treatment latency" OR "latency to treatment" OR "symptom overlap" OR "symptom recognition" OR "symptom appraisal" OR "symptom attribution" OR "symptom misattribution" OR "symptom perception" OR "symptom awareness" OR belief* OR attitude* OR heuristic* OR "cognitive bias*" OR "coping style*" OR "self regulation" OR screening OR "help seeking " OR "information seeking" OR DE "Help Seeking Behavior" OR DE "Health Literacy" OR DE Self-Efficacy OR "Self-efficacy" OR "self medication" OR "self treatment" OR stigma OR DE Health Care Access OR "access to care" OR "access to treatment" OR "access to medication" OR affordab* OR "waiting time" OR polarity OR underreporting OR "under reporting" OR "therapeutic alliance" OR "clinical competenc*" OR "diagnostic error*" OR "diagnostic valid*" OR "diagnostic reliability" OR "diagnostic criter*" OR "diagnostic accuracy" OR "stability of diagnosis" OR "diagnostic stability" OR misdiagnosis OR DE Self Referral OR DE Patient Referral OR "Referral*" OR "Consult*" OR insight OR DE "Health Attitudes" OR "attitude to health" OR DE Treatment Compliance OR adherence OR compliance OR implementation OR policy |
|  | |
| **EBSCOHost-CINAHL** | |
| **Illness**  Terms: 6 | MH "Bipolar Disorder" OR bipolar disorder OR mania OR manic OR hypomania OR hypomanic |
| AND | |
| **Age group**  Terms: 10 | MH "Adolescent Health" OR adolescen* OR MH "Emerging Adulthood" OR young adult* OR MH "Pediatrics" OR pediatric* OR paediatric* OR youth* OR teen* OR juvenile* |
| AND | |
| **Components of delay**  Terms: 59 | "duration of untreated" OR "delayed diagnosis" OR "diagnostic delay" OR "delay in treatment" OR "treatment latency " OR "latency to treatment" OR "symptom overlap" OR "symptom recognition" OR "symptom appraisal" OR "symptom attribution" OR "symptom misattribution" OR "symptom perception" OR "symptom awareness" OR belief* OR attitude* OR heuristic* OR "cognitive bias*" OR "coping style*" OR "self regulation" OR screening OR "help seeking " OR "information seeking" OR MH "Help Seeking Behavior" OR MH "Health Literacy" OR MH Self-efficacy OR "Self-efficacy" OR "self medication" OR "self treatment" OR stigma OR MH Health Care Access OR "access to care" OR "access to treatment" OR "access to medication" OR affordab* OR "waiting time" OR polarity OR underreporting OR "under reporting" OR "therapeutic alliance" OR "clinical competenc*" OR "diagnostic error*" OR "diagnostic valid*" OR "diagnostic reliability" OR "diagnostic criter*" OR "diagnostic accuracy" OR "stability of diagnosis" OR "diagnostic stability" OR misdiagnosis OR (MH "Referral and Consultation") OR referral* OR consult* OR insight OR "MH "Attitude to Health" " OR "attitude to health" OR MH "Medication Compliance" OR adherence OR "compliance" OR implementation OR policy |

Forward slashes indicate MeSH Terms in MEDLINE and EMBASE searches; DE and MH indicates subject headings in PsycINFO and CINAHL, respectively. Differences in search term counts reflect differences in MeSH/subject headings available to respective databases.

**Supplementary Table 2: Studies meeting scoping review inclusion criteria**

| **Study ID*** | **Study Aims** | **Study Design; Sample Size** | **Recruitment Procedure** | **Inclusion and Exclusion Criteria** | **Average age** (mean (y) +/- SD unless otherwise specified) | **Onset Age**  (mean (y) +/- SD unless otherwise specified) | **Gender/ Sex** | **Ethnicity** | **Socioeconomic Context** | **Proportion with BD** | **Diagnostic definition;**  **Interview/Scale Instrument** | **Comorbidities** | **Country; Clinical Setting; Population Density Setting** |
| --- | --- | --- | --- | --- | --- | --- | --- | --- | --- | --- | --- | --- | --- |
| Suppes 2001(51) | Characterize an initial cohort of the Stanley Foundation Bipolar Network (SFBN) | Cross-sectional study  261 | Referred from within each site’s clinic or new patients joining the clinic. In many cases, patients were referred by local physicians or by self-referral from local advocacy groups Patients could receive their psychiatric medical care entirely within each clinic, or see a different (non-Network) psychiatrist and be followed monthly in consultation and evaluation with the SFBN psychiatrists. | Inclusion: history of a bipolar disorder meeting DSM-IV criteria for bipolar I, bipolar II, bipolar disorder NOS, or schizoaffective disorder, bipolar type.  Exclusion: none other than current substance abuse requiring treatment in another setting or being unwilling to attend monthly visits as part of the naturalistic long-term follow-up study, participate in detailed evaluations and prospective life charting, and consider entering open and controlled clinical treatment trials if they became symptomatic in their course of illness | 43 +/- 1 | Age of first mood episode: 23 +/- 10  First symptoms: 20 +/- 11 | 56% female gender | 93% Caucasian 2% African American 5% Other | Employment:  Full-time 25%,  Part-time 9%; Unemployed 6%; Volunteer 5%;  Unable to work 21%; Retired 5%;  Other 10%;  Unknown 1%  Education:  <high school graduate 2%;  high school graduate 5%;  some post high school 38%; at least a college graduate 55%  Household income: <$20,000 39%;  $20,000-$39,999 23%; $40,000-$79,999 25%; $80,000+ 13%  Occupational functioning limited by bipolar illness: Not limited 18%;  Mildly 20%;  Moderately 22%; Markedly 22%;  Severely 18% | 99%:  BD1 81% BD2 16% BD-NOS 2% | DSM-IV  SCID | Lfetime Axis 1 disorder 67%  substance abuse disorder 41%  anxiety disorder 44%  eating disorder 15%  impulse control disorder 10% | USA, Germany, Netherlands  General Outpatient  Population density setting not specified |
| Fleck 2002(50) | Examine use of antipsychotics in African Americans with affective illness. | Cohort study  58 | Recruited consecutive patients admitted with manic or mixed episode | Inclusion:  (1) DSM-IV manic or mixed episode; (2) 15 - 45 years of age; (3) no prior hospitalizations; (4) less than 1 month of prior psychotropic medication; (5) ability to communicated in English; (6) living within 50 miles of Cincinnati, Ohio. Additionally, only white and African American patients were included.  Exclusion:  (1) psychiatric symptoms due to acute medical illness or due to (2) acute intoxication/withdrawal; or (3) IQ<70. This analysis includes patients who completed at least 4 months of follow-up (7 patients lost to follow-up) | Not specified though related publication (PMID 11018221) on first n = 50 consecutively enrolled participants cited age of  25 +/- 8 | Not specified though related publication (PMID 11018221) on first n = 50 consecutively enrolled participants cited onset age of  22 +/- 8 | Not specified though related study (reference 20, Strakowski et al 2000) on first n = 50 consecutively enrolled participants cited 36% female gender | 59% White,  41% African American | Not specified though related study (reference 18, Strakowski et al 2000) on first n = 50 consecutively enrolled participants cited mean 12 years of education +/- 3 years and 17% employment. | 100% | DSM-IV  SCID | Not specified | USA;  General Inpatient & General Outpatient  Population density setting not specified |
| Goldberg 2002(34) | Evaluate whether delayed treatment of bipolar disorder with mood stabilizers was associated with poorer functional outcomes and a greater number of lifetime suicide attempts, and to identify features associated with prolonged time delays from affective symptom onset until (1) the diagnosis of bipolar disorder and (2) initiation of a first mood stabilizer | Cross sectional study  56 | Consecutively evaluated clinic patients. | Inclusion:  Diagnosis of bipolar I, bipolar II, or bipolar NOS.  Exclusion:  Subjects for whom symptom history or comprehensive medication histories could not be ascertained with reasonable confidence were not included in the study group. | 41 +/- 13 | Symptom onset age at 22 +/- 10  Bipolar illness was diagnosed at a mean age of 32 +/- 12 | 38% female; gender/sex not specified | 77% White | Not specified | 100% | DSM-IV  SCID | Lifetime alcohol abuse or dependence 32% | USA  Specialized Outpatient  Population density setting not specified |
| Dilsaver 2005(44) | Ascertain the rate of bipolarity among adolescent Hispanic youths referred for the treatment of major depressive disorder (MDD) in a community mental health clinic (CMHC) for the destitute on the border of Mexico and the United States. | Cross sectional study  49 | The threshold for referral to the clinic is moderate to severe impairment in multiple domains; Primary referral sources included school disciplinary authorities and juvenile courts | Inclusion:  between ages 12 and 17  Exclusion:  non-hispanic youth | 15 +/- 2 | Not specified | 67% female; gender/sex not specified | 100% Hispanic | The per capita income in this community is $4500. | 55% (BD-I 8%; BD-II 6%; mixed state 41%) | DSM-IV  SCID | Drug abuse 25%  “high prevalence” of "conduct problems" | USA  General Outpatient  Population density setting not specified |
| Kessing 2005(36) | Investigate the diagnostic stability of the ICD-10 diagnosis of mania/bipolar disorder as made by clinicians within psychiatry and to estimate the gender and age associations with time to first diagnosis of bipolar disorder. | Population study  4116; 68 diagnosed with mania/ bipolar disorder at first contact when aged <20 | Data was pulled from the Danish Psychiatric Central Research Register for any patients with a main diagnosis of manic episode or bipolar affective disorder | Inclusion:  All outpatients and inpatients with at least one main diagnosis of mania/bipolar disorder during the study period | Not Specified | Not Specified | 60% female gender | Not Specified | Not Specified | 100% (having received a diagnosis of mania or bipolar disorder at least once) | ICD10  Not specified | Not specified | Denmark  Patient Registry/ Population Data (inpatient & outpatient)  Population density setting not specified |
| Bhargava Raman 2007(45) | Estimate the lifetime prevalence of bipolar II disorder in children and adolescents presenting with DSM-IV major depressive disorder (MDD) | Cross sectional study  61 | 61 consecutive subjects without a baseline diagnosis of bipolar disorder aged <18 years recruited from a major psychiatric hospital; 95% self-referred. | Inclusion:  A baseline DSM IV diagnosis of MDD.  Exclusion:  history of substance use | Not specified | 13 +/- 2 | 46 % female gender | Not specified | Attending school 51%  Not attending school 48%  Never attended school 2% | 33% | DSM-IV with Reduced threshold for hypomania to 2 days  MAGIC | Dissociative motor disorder 25%  Specific learning disability 18%  OCD 5% | India  Specialized Outpatient  79% Urban  21% Rural |
| Suominen 2007(30) | To obtain a comprehensive view of differences between bipolar disorder (BD) patients with onset at early versus adult age in a representative study cohort | Cross sectional study  191 | All in- and out-patients in the catchment area of Jorvi Hospital with a possible new DSM-IV BD episode during the study period using the Mood Disorder Questionnaire (MDQ). After a positive screen or on clinical suspicion of BD, the subject was informed about the study protocol and written informed consent was requested. | Inclusion:  Referred for assessment, and seeking treatment;   Already received care and was now showing signs of deteriorating clinical state (or change in mood in case of hypomania);  Screened positive on MDQ; or if screened negative, then a health care professional still suspected a bipolar disorder;  The final study group included in the analyses consisted of DSM-IV bipolar I and II patients with a current episode.  Exclusion: A clinical diagnosis of ICD-10 schizophrenia | 38 +/- 12 for the whole group;  31 +/- 12 for the early onset subgroup (defined as less than 18 years of age)  41 +/- 11 years for the late onset subgroup (defined 18 years or older) | 24 +/- 10 for the whole group  14 +/- 3 for the early onset subgroup (defined as less than 18 years of age)  28 +/- 9 years for the late onset subgroup (defined 18 years or older) | 53% female gender | Not Specified | 42% Married or cohabiting, 32% Not cohabiting,  26% Divorced or widowed; 16% University education, 22% College,  22% Vocational,  40% No professional education; 54% Employed,  10% Student,  13% Unemployed,  32% Disability pension | 100%  (BD-1 47%;  BD-II 53%) | DSM-IV  SCID | Lifetime history of any psychiatric comorbidity of 84%, including  Anxiety Disorder 53%,  Substance dependence/  abuse 51%  Personality disorder 43%  Eating disorder 16% | Finland  General Inpatient; General Outpatient  Population density setting not specified |
| Berk 2007(35) | To investigate the history of illness of people with BD-I or SCZA diagnosis. Outcomes from the Bipolar Comprehensive Outcomes Study (BCOS) | Cohort study  218 | Through public hospital system and advertisements in local print media. | The study inclusion criteria were: a primary diagnosis of bipolar I disorder or schizoaffective disorder; at least 18 years of age; a level of understanding and ability to communicate in English sufficient to complete study instruments; willing to comply with the requirements of the study; contactable by phone or mail; and prescription for at least one of the following mood stabilisers at baseline (as monotherapy or in combination): olanzapine, lithium carbonate, sodium valproate or carbamazepine.  Exclusion: diagnosis of schizophrenia; organic psychosis or dementia; involved in a controlled clinical trial 30 days prior to the study or at any time during the study. | 42 +/- 13 | First depressive episode at median age of 21 (IQR 17-29)  First manic episode at median age of 24 (IQR 19-31)  Median age of diagnosis (Bipolar 1 or Schizoaffective) at 30 (IQR 23-37) | 57% female; gender/sex not specified | Not specified | Not specified | 75% | DSM-IV-TR  MINI | Not specified | Australia  Clinical Setting not Specified  Mixed Urban (Melbourne)/Rural (Geelong) |
| DelBello 2007(49) | Examine the 12-month outcome of bipolar adolescents following an initial hospitalization for a manic or mixed episode. | Cohort study  71 | Adolescents admitted for their first hospitalization for bipolar disorder, type I, manic or mixed were recruited from consecutive inpatient admissions between July 1, 1999 to Dec. 1, 2003. | Inclusion: - 12-18 years old - DSM-IV criteria for a current manic or mixed episode  Exclusion: - no prior treatment with anticonvulsants, antidepressants, or antipsychotics.  - “mental retardation” (IQ<70) or a manic or mixed episode resulting entirely from an unstable medical or neurological disorder or acute intoxication or withdrawal from drugs or alcohol, as determined by symptom resolution within 72 hours. | 15 +/- 2 | Not specified | 41% female sex | 76% Caucasian | Average Hollingshead Scale rank of 3  +/- SD = 2 | 100% | DSM-IV  K-SADS | ADHD 44%;  Disruptive Behaviour Disorder 51%;  Anxiety Disorder 23%;  AUD 8%;  Cannabis UD 7% | USA  General Inpatient  Population density setting not specified |
| Riyami 2009(29) | To characterize health services utilization in Oman and the barriers among school going adolescents and youths with DSM IV disorders | Cross sectional study; Other: Survey  17 | The survey adopted a nationally representative, multi-stage, stratified random sampling design to select its subjects.  The latest data obtained from the Ministry of Education, Oman was used as the sampling frame. | Not specified | Not specified; participants were between 14-23 years of age. | Not specified | Not specified | Not specified | Not specified | 100% | DSM-IV  WMH-CIDI | Not specified | Oman  Patient Registry/ Population Data  Population density setting not specified |
| Soutullo 2009(41) | To describe the clinical characteristics and symptoms of children with BD prior to their diagnosis and at the time of diagnosis in a sample in Spain | Cross sectional study  38 | Retrospectively reviewed the charts of all children and adolescents with diagnosed BD evaluated in an outpatient clinic sample from 1999 to 2005. | Inclusion criteria:  age 18 years old,  a DSM-IV-based diagnosis of BD,  ability to communicate with the medical team.  Exclusion criteria:  Symptoms of BD due to a medical condition or due to a substance. | On initial clinic consult,  Median age of 14 (IQR: 13 - 15) | Median age of symptom onset: 12 (IQR: 9 - 12) Median age of diagnosis 14 (IQR: 11-16) | 21% female sex | 92% Caucasian; 3% African 2.6%; 3% Hispanic;  3% Arabic | Median 8 years of education (IQR 6-9) with 42% repeating a grade; 18% having left school; 21% requiring tutoring;  11% requiring academic accommodations. | 100%;  45% children had a diagnosis of BD1,  5% had BD2  50% had BP-NOS, of which the majority lacked episode duration, 32% had BD precipitated by SSRI use,  and 5% had recurrent irritability with first degree family history. | DSM-IV  K-SADS | Comorbidities prior to diagnosis of BD:  ADHD 21%  Major depression 18%  ODD 16%  Mood disorder NOS 11%  Separation anxiety 11%  CD 11%  SUD 11%  Cyclothymia 3%  Comorbidities at time of diagnosis:  ADHD 21%  SUD 18%  CD&ODD 16%  Anxiety Disorder 16% | Spain  Specialized Outpatient  Urban 63%  Small to mid-size towns 37% |
| Kozloff 2010(31) | Measure the lifetime prevalence of BD, and to describe the sociodemographics, comorbidities, and use of mental health services among 15-24 year olds with BD. | Population study  191 | Random selection from private households | Community dwelling | Not specified; age range 15-24 | Not specified | 56% female sex | 76% White | Low Income Adequacy 18%  Full-time student 41%  Employed full-time 57% | 100% | DSM-IV  WMH-CIDI | Lifetime anxiety disorder 47%  Substance use disorder in prior 12 months 42% | Canada  Community mental health survey  Rural 22% |
| Evans-Lacko 2010(37) | Examine longitudinal patterns of complexity, continuity, and initiation of treatment for youth diagnosed with bipolar disorder; explore bipolar diagnosis stability and its relationship to observed treatment patterns. | Other: Administrative Data  426 | Data was derived from a cohort of privately insured youth (ages 6-18) diagnosed with bipolar disorder was identified from the 2000-2001 Thomson/Medstat-MarketScan database | Inclusion: Received diagnosis of bipolar disorder on at least 2 outpatient visits 1 inpatient hospitalization.  Exclusion: Receiving any type of bipolar disorder diagnosis during the first 4 months of the 24-month study period (to ensure study of new rather than pre-existing diagnoses);  Enrolled for less than six additional months following their initial diagnosis of bipolar disorder;  Co-occurring seizure disorders (may have received mood stabilizing medications for reasons other than treatment of bipolar disorder). | 14 | Not specified | 48% female gender | Not specified | Not specified | 100% | ICD9  A uniform standardized instrument was not specified. | Not specified | USA  Mixed clinical settings  Sub/urban 88% Rural 12% |
| Evans-Lacko 2011(38) | Investigate patterns of mental health services, psychotropic treatments, and psychiatric diagnoses received by youth diagnosed with bipolar disorder. | 323 | Data was derived from a cohort of privately insured youth diagnosed with bipolar disorder was identified from the 2000-2001 Thomson/Medstat-MarketScan database. | Inclusion: Received diagnosis of bipolar disorder on at least 2 outpatient visits 1 inpatient hospitalization.  Exclusion:  Enrolled for less than six additional months following their initial diagnosis of bipolar disorder;  Co-occurring seizure disorders (may have received mood stabilizing medications for reasons other than treatment of bipolar disorder). | 14 +/- 3 | Not specified | 52% female gender | Not specified | Not specified | 100% | ICD9  A uniform standardized instrument was not specified. | (at time of initial diagnosis:)  Depression 45%  ADHD 20%  CD/ODD 12%  Adjustment reaction 7%  Personality disorder 7%,  Mood NOS 7%  Schizophrenia 3%  ASD 1%  Substance abuse 1% Eating disorder 0.3% | USA  Mixed clinical settings  88% sub/urban, 12% rural |
| Chilakamarri 2011(40) | To compare research-assessment validated diagnoses with prior clinician diagnoses in order to identify rates of underdiagnosis, overdiagnosis, or both of BD compared to ADHD and MDD. | Cross sectional study | Not specified; 64 patients seen initially by a single clinician in a "community primary care mental health setting" | Not specified; age 7 - 18, inpatients and outpatients, diagnosed with ADHD, BD, or MDD | 14 +/- 3 | Age of onset of mania 11 +/- 3; Age of onset of depression 10 +/- 3 | 52% female sex | 90% Caucasian, 10% African American | Not specified | 33% | DSM-IV  A uniform standardized instrument was not specified | Not specified | USA  General Outpatient  Population density setting not specified |
| Crowe 2011(27) | Examine parental views on the onset of symptoms, impact on functioning and meanings attributed to their child’s bipolar disorder. | Qualitative research  85 | Youth in the core psychotherapy study (PMID: 25346391) were recruited by referral  Caregivers were then approached by a research nurse to complete a written self-report of their perceptions. Participants were recruited and responded between 2003 and 2009 | Youth included in the core psychotherapy study were diagnosed with BD.  Caregivers included in this study were identified by the youth as having known her/him while growing up. | Mean age of diagnosed youth at entry to core study = 27; SD not specified; range 15-34 | Onset of depressive symptoms by age 16 in 61%  (mostly 13-16).  Onset of manic symptoms occurred by age 19 in 50% | 76% female (gender/sex not specified) | Not specified | Not specified | Study was of caregivers of youth; 100% of youth a diagnosis of BD:  78% BD1,  17% BD2,  5% BD-NOS | Diagnostic criteria and a uniform standardized instrument were not specified | Index youth:  Any anxiety disorder 58%;  Panic Disorder 41%  Social Phobia 19%  SUD 50%  Eating Disorder 30%  Personality disorder 43% | New Zealand  Mail-in responses from parents of youth participating in outpatient psychotherapy study  Population density setting not specified |
| Dusetzina 2011(47) | Examine the extent to which children with BD1 receive recommended treatment of mood-stabilizer or second-generation antipsychotic monotherapy and factors associated with its receipt | Other: Administrative/ Insurance claims data  412  (297 aged 13-17) | Longitudinal, retrospective person-level cohort study of commercial claims data collected by Thomson Reuters MarketScan from January 1, 2005, through December 31, 2007. MarketScan includes deidentified data on clinical utilization and expenditures for inpatient, outpatient, and pharmacy services for approximately 100 privately insured payers within the United States. | Inclusion: - Ages 6 to 17  - At least one inpatient or two outpatient claims for BD1 manic or mixed episode type  - Continuously insured for 180 days before and one year after their index date of diagnosis  Exclusion:  - schizophrenia, pervasive developmental disorders, mental retardation, or substance abuse disorders in the 180 days before their BD1 diagnosis.  - medical conditions that mimic symptoms of mania, such as temporal lobe epilepsy, multiple sclerosis, hyperthyroidism, closed or open head injury, and systemic lupus erythematosus, or conditions that complicate the treatment of bipolar disorder, such as pregnancy, were also excluded - having filled an antipsychotic, anticonvulsant, or lithium during the 180 days before the index diagnosis (to ensure new diagnoses only) - hospitalized within 60 days before or 45 days after the index bipolar diagnosis as prescription claims did not include medications received in hospital  - insurance plans that did not provide information about medication use | Not reported; 72% aged 13-17 | Not specified | 53% Female; Sex;   Not specified for 13-17 category | Not specified | Not specified | 100% | ICD9  A uniform standardized instrument was not specified | Among full sample (not specified to BD1 cohort):  ADHD 22%;  Depressive disorder 18%;  Disruptive behaviour disorder 10%;  Anxiety disorder 6% | USA  Mixed clinical settings  Population density setting not specified |
| Fontanella 2015(48) | Examine rates of conformance to treatment guidelines for children and adolescents with new episodes of BD Identify patient demographic, clinical and provider characteristics associated with guideline-concordant care | Cohort study; Other: Administrative Data  4047 | Data was derived from Medicaid eligibility and claims files obtained from the Ohio Department of Jobs and Family Services; All children and adolescents aged 5-18 years with a new episode of BD in 2006-2010 who were continuously enrolled in Ohio’s Medicaid program for a 13 month period (at least 6 months prior to and 7 months after the first claim for BD). | Inclusion:  - Age 5-18 years  - New BD episode  Exclusion:  - Schizophrenia, ASD, and “mental retardation”  - Pregnancy  - Seizure disorder and on an anticonvulsant.  - Inpatient stay of greater than 15 days during the seven month treatment period as medications could not be captured during hospitalization. | 15 +/- 3 | Not specified | 50% Female gender | 73% White;  27% Non-White | Poverty 76%;  Disability 16%;  Foster Care 8% | 100% | ICD9  A uniform standardized instrument was not specified | Conduct disorder 6%;  ADHD 6%  Substance Abuse Disorders 4%;  Anxiety Disorders 2%;  "Select" medical conditions 5% | USA  Mixed clinical settings  77% Urban,  22% Rural |
| Kessing 2015(39) | Investigate the diagnostic stability of the ICD-10 diagnosis of pediatric mania/bipolar disorder | Population study  354;  41% were diagnosed on first contact whereas the remaining patients were diagnosed on follow-up. | Data was derived from a nation-wide with registration of all psychiatric hospitalizations and ambulatory care in Denmark (The Danish Psychiatric Central Research Register; DPCRR). | Age <19 years;  At least one main diagnosis of mania/bipolar disorder during the study period from January 1, 1994 to December 31, 2012. | Of patients with BD diagnosis at first contact: Median age at first contact 17; IQR 16-18.  Not specified for remainder of sample, though younger than 19 at time of BD diagnosis. | Not specified | Of patients with BD diagnosis at first contact:  51% female sex.  Not specified for remainder of sample. | Not specified | Not specified | 100% | ICD10  A uniform standardized instrument was not specified | Not specified | Denmark  Patient Registry/ Population Data  Population density setting not specified |
| Daigneault 2015(43) | Evaluate the consistency between initial suspicious of bipolar disorder from general practitioners and the diagnosis made by shared-care psychiatrists. | Cross sectional study  538  (sample size for 18-25 age cohort not given). | Chart review of patients referred by a general practitioner for assessment by a psychiatrist from 1998 to 2010 at the hospital clinic, | Inclusion:  Have a specific reason for referral.  Diagnosed with BD.  No exclusions | 40 (+/- 12); focused on data from 18-25 year old cohort | Not specified | 58% female gender | Data not specified for bipolar sub-sample, though the catchment area for this clinic (n = 372, 268) is 60% Caucasian;  19% Arab;  15% Asian;  15% Black/ African;  6% Hispanic/ Latino | Not specified | Data focused on patients diagnosed with BD at the clinic, which were 6.1% of all referred patients to the clinic. Of these, 40% BD1;  41% BD2;  12% Cyclothymic Disorder;  7% BD NOS. | DSM-IV  A uniform standardized instrument was not specified | Not specified | Canada  General Outpatient; Primary Care  Urban |
| Assad 2015(33) | To assess the rate of bipolar affective disorder (BAD) patients seeking traditional healers, the sociodemographic and clinical correlates of those patients | Cross sectional study  Subset of 299 diagnosed with BD and data on contact with traditional healers | Patients from 3 psychiatric hospital in Cairo with provisional diagnosis of BAD referred to study group | Inclusion criteria not detailed  Exclusion:  Medical comorbidities | 36 +/- 11 and  34 +/- 12 in those with and without history of traditional healers, respectively | 23 +/- 6 and  24 +/- 8 in those with and without history of traditional healers, respectively | 45% female gender | Ethnicity not provided  Of those with data on seeking traditional healers,  95% Muslim  5% Christian | Of those with data on seeking traditional healers:  Illiterate 8%  Basic education 31% Secondary education 40%  University education 21% | 88% of complete sample  84% BD1  4% BD2  Data on subsample of 100% BD also presented | DSM-IV-TR  SCID | Not specified | Egypt  Patient setting and population density setting not specified |
| Kvitland 2016(53) | Examine the associations between Duration of Untreated bipolar and key clinical outcomes at baseline in BD1, and at one year follow-up, and to evaluate the influence of cannabis use. | Cohort study; Cross sectional study  101 initially included,  62 participated in 1 year follow-up. | Patients coming to their first treatment for BD1 were recruited consecutively from 2003 to 2013, from in- and outpatient units at all major hospitals in the Oslo area;  Recruitment was part of the larger Thematically Organized Psychosis (TOP) Study at the University of Oslo and Oslo University Hospital. | Inclusion:  - Diagnosis of BD Type 1 - Age 17-65.  Exclusion:  - Patients were excluded if they had pronounced cognitive deficits (IQ < 70)  - Unable to speak a Scandinavian language or give written informed consent.  - For the current first-treatment study they could not have a previous diagnosis of BD, or have received adequate treatment for BD in the form of lithium, anticonvulsants or antipsychotics. | 31 +/- 10 | Any polarity: 24 +/- 10  Depressive Episode 23 +/- 10  Hypomanic Episode 23 +/- 7  Psychotic Episode 26 +/- 9  Mixed Episode 27 +/- 6  Manic Episode 29 +/- 11 | 60%female  (not specified as gender or sex) | 84% Caucasian | Mean education 14 +/- 2 years;  Single 53%;  Unemployed 50% | 100% | DSM-IV  SCID | SUD 27%  (of which 82% was cannabis use disorder) | Norway  Specialized Inpatient; Specialized Outpatient  Population density setting not specified |
| Ribeiro-Fernandez 2019(42) | 1) evaluate the longitudinal diagnostic stability of DSM-IV BD (I, II, or NOS) in children and adolescents 2) describe the longitudinal stability of BD subtypes, especially BD-NOS  3) identify premorbid or baseline clinical characteristics that may predict the evolution from BD-NOS to BD-I or BD-II. | Cross sectional study; Other: Chart Review  72 | Retrospectively evaluated patients diagnosed with BD from January 2000 to December 2014 in the Child & Adolescent Psychiatry Unit. | Inclusion:  DSM-IV or DSM-IV-TR diagnosis of BD;  Age less than 18 years at the time of diagnosis;  Ability to communicate with the medical team.  Exclusions:  Patients with neurological disorders or symptoms;  other medical conditions, schizophrenia, or intellectual developmental disorder (IQ < 70) | Median [IQR]: Age at first consult with the clinic 14 [10-16],   BD Diagnosis 15 [11-16] | Median [IQR]: First symptom 11 [7-14] | Female 25%; Male 75% (Sex) | 94% Caucasian;  3% Arab;  1% African;  1% Hispanic | 14% Adopted | 100% | DSM-IV; DSM-IV-TR  K-SADS | 75% of patients had at least one comorbid disorder 38% had two or more comorbid disorders.  The most frequent psychiatric comorbid disorders were:  ADHD 47%;  ODD or CD 20%;  SUD 13%  Anxiety disorders 7%, Eating disorders 7%,  OCD 7%;  4% other. | Spain  Specialized Outpatient  58% Urban, 42% Suburban |
| Post 2020(46) | Compare the age at which bipolar disorder onset & treatment occur in the US and in Europe. | Cross sectional study  968;  675 from US, 292 from Europe | Recruited from advertisements and local clinics in four cities in the United States (Los Angeles, Dallas, Cincinnati, Bethesda) and three in Europe (Utrecht, the Netherlands and Freiburg and Munich, Germany) from 1995 to 2002 as part of the Stanley Foundation Bipolar Treatment Outcome Network (SFBN). | Poorly described even in cited study.  Inclusion: All participants participated in an outpatient research network (the Stanley Foundation Bipolar Treatment Outcome Network (SFBN)) Exclusion: Current active substance abuse requiring treatment at another facility, or severe comorbid medical problems. | 41 (variance not specified) | US: 17 +/- SEM = 0.4  Europe: 24 +/- SEM = 0.6 | US: 58% female gender  Europe: 54% female gender | US: 90% White  Europe: 96% White | Not specified | 100%;  75% BP I,  23% BP II,  2% BP-NOS | DSM-IV  SCID | US:  Anxiety Disorder 47%, Alcohol Abuse 33%; Substance Abuse 38%  Europe:  Anxiety Disorder 28%;  Alcohol Abuse 15%;  Substance Abuse 18% | USA, Netherlands, Germany  Patient setting and population density setting not specified |
| Carr 2023(32) | Whether Maori (Indigenous people of New Zealand) and non-Maori in first episode diagnoses persist over time and how these differences impact service use. | Population study  2316 | Anonymized routine mental health service data | Inclusion: A diagnosis of schizophrenia, bipolar disorder 1, psychotic depressive disorder, substance-induced psychotic disorder, schizoaffective disorder, organic psychotic disorder, other psychotic disorder, or a non-specific psychotic disorder; with a first episode of psychosis between 2009-2012, aged 13-25 at the time of the first recorded psychosis-related diagnoses. Individuals were required to be able to be linked to other datasets through the Integrated Data Infrastructure spine.  Exclusion (based on cited study): a provisional diagnosis only and no further psychosis-related diagnosis; no face-to-face contacts or inpatient treatment during the three months prior and three months post diagnostic period (based on the assumption that face-to-face contact is required for FEP diagnosis); and overseas travel for more than half of the time between the start of PRIMHD and the end of the diagnosis period (as a prior diagnosis could not confidently be ruled out). | Not specified | Cited study reports mean age of diagnosis of FEP (not specific to BD) as 20 | Cited study reports 35% and 38% female gender for Maori and Non-Maori youth, respectively. | 59% Non-Maori  41% Maori | Cited study reports NZ Deprivation Index in quintiles, with highest quintile indicating greater deprivation  Maori:  1 (less deprivation): 6%  2: 7%  3: 13%  4: 22%  5 (more deprivation): 52%  Non-Maori:  1 (less deprivation): 15%  2: 19%  3: 20%  4: 22%  5 (more deprivation: 24% | At the five-year period from FEP: 28% in non-Maori; 18% in Maori | DSM-5; ICD10  A uniform standardized instrument was not specified | Among those with an initial diagnosis of affective psychosis  Maori:  Moor or Anxiety: 39%  SUD: 38%  Non-Maori:  Moor or Anxiety: 47%  SUD: 24% | New Zealand  Specialized Outpatient  Cited study reports:  Maori:  Urban 43% Semi-Urban 29% Rural 27%  Non-Maori:  Urban 59% Semi-Urban 23% Rural 27% |
| Brancati 2023(52) | To examine differences between patients with bipolar I and II disorders with particular emphasis on the early phases of the disorders | Cohort Study  100 | Four cohorts of patients recruited between April 1994 and March 2022 were gathered from different datasets of patients followed longitudinally, in most instances for 10 or more years. | Inclusion: bipolar I or bipolar II disorder | 46 +/- 13 | 24.7  (Median onset 20-22) | 60% female sex; BD1 57%,  BD2 67% | Not specified | BD1:  Single 34%;  Married 42%;  Divorced 23%;  Widowed 2%  Work full-time 22%  Work part-time 9%  Unemployment insurance 9%  Social assistance 13%  Disabled 28%  Other 6%  Retired 8%  Student 7%  BD2:  Single 18%;  Married 58%;  Divorced 21%;  Widowed 4%  Work full-time 15%  Work part-time 7%  Unemployment insurance 7%  Social assistance 13%  Disabled 24%  Other 11%  Retired 8%  Student 8% | 100%:  73% BD1  27% BD2 | DSM-IV  SCID; SADS-L; DIGS | BD1:  SUD 35%  Generalized Anxiety 30%  Panic Disorder 21%  Social Anxiety 20%  Personality Disorders 12%  OCD 11%  Primary Insomnia 10%  ADHD 5%  Learning Disability 5%  BD2:  Generalized Anxiety 34%  SUD 27%  Social Anxiety 25%  Panic Disorder 24%  Primary Insomnia 19%  Personality Disorders 13%  OCD 14%  ADHD 6%  Learning Disability 6% | Canada (Majority), Germany, Czech, Sweden, Denmark, Austria  Specialized Outpatient  Patient population density not specified |
| Singhai 2024(28) | To analyze the sociocultural influences that impact treatment adherence and help-seeking behaviors among individuals diagnosed with BD and MDD who received care in a tertiary care psychiatry unit | Cross sectional study  33 | A purposive sampling technique of individuals who received care in a tertiary care psychiatry unit situated in an academic hospital located in Western Rajasthan, India. The number of participants was based on data saturation.  The interviews also included evaluation of the sociocultural beliefs of a key informant (interviewed separately) i.e., a family member or a caregiver of the patient, who was knowledgeable about clinical problems and life circumstances of the patient and lived with the patient as well as involved in his care and treatment maintenance for at least one year before enrollment in the present study. | Inclusion: Non-adherent (MARS score <6) 18-60 years old who understood English or Hindi and met DSM-5 criteria of unipolar depression or BD currently in remission.  Key informants that spoked English or Hindi were also included.  Exclusion:  Patients with other comorbid mental disorders (except tobacco use disorder), severe medical illnesses, cognitive disorders, or intellectual disabilities.  Key informants who had severe medical/surgical illness or mental illnesses (except tobacco use disorder). | 29 +/- 8 | 21 +/- 4 | 40% female gender | Not specified | Married 40%  Unmarried 20%  Separated 20%  Illiterate 20%  Primary School 30%  High School 40%  Graduate and Above 10% | 33% Bipolar Disorder (type not specified) | DSM-5  CFI (for assessment, not diagnosis) | Not Specified | India  Specialized Inpatient  Rural 60% |

*First author’s last name and year of publication
ADHD: Attention-Deficit/Hyperactivity Disorder
AUD: Alcohol Use Disorder
BD1: Bipolar I Disorder,
BD2: Bipolar II Disorder,
BD-NOS: Bipolar Disorder Not Otherwise Specified,
BD: Bipolar Spectrum Disorder
SUD: Substance Use Disorder

**Supplementary Table 3: Intersection of interval and factors investigated in included studies.**

|  |  | Interval | | | | |
| --- | --- | --- | --- | --- | --- | --- |
|  |  | Appraisal | Help-Seeking | Diagnostic | Pre-Treatment | Not Specified |
| Factors | Individual | 1 | 4 | 7 | 7 | 0 |
|  | Family | 0 | 1 | 2 | 0 | 0 |
|  | Socioeconomic | 1 | 4 | 1 | 4 | 0 |
|  | Other Patient | 0 | 0 | 0 | 0 | 0 |
|  | Disease | 0 | 1 | 7 | 4 | 0 |
|  | Comorbidity | 1 | 1 | 1 | 6 | 0 |
|  | Other Disease | 0 | 0 | 2 | 2 | 0 |
|  | Clinician | 0 | 0 | 6 | 3 | 0 |
|  | Health System | 0 | 0 | 2 | 2 | 0 |
|  | Other Systemic | 0 | 1 | 0 | 3 | 0 |

Numbers reflect how many studies from those included in our review (total n = 27) contained data on the respective intersection of patient, disease, and systemic factors in the appraisal, help-seeking, diagnostic, and pre-treatment intervals.
